# The cost of emergency hospital admissions for falls on snow and ice in England during winter 2009/10: a cross sectional analysis

**DOI:** 10.1186/1476-069X-10-60

**Published:** 2011-06-17

**Authors:** Caryl Beynon, Sacha Wyke, Ian Jarman, Mark Robinson, Jenny Mason, Karen Murphy, Mark A Bellis, Clare Perkins

**Affiliations:** 1 North West Public Health Observatory, Liverpool John Moores University, Henry Cotton Building, 15-21 Webster Street, Liverpool, L3 2ET, UK

## Abstract

**Background:**

In the UK, the 2009/10 winter was characterised by sustained low temperatures; grit stocks became depleted and surfaces left untreated. We describe the relationship between temperature and emergency hospital admissions for falls on snow and ice in England, identify the age and gender of those most likely to be admitted, and estimate the inpatient costs of these admissions during the 2009/10 winter.

**Methods:**

Hospital Episode Statistics were used to identify episodes of emergency admissions for falls on snow and ice during winters 2005/06 to 2009/10; these were plotted against mean winter temperature. By region, the logs of the rates of weekly emergency admissions for falls on snow and ice were plotted against the mean weekly temperature for winters 2005/06 to 2009/10 and a linear regression analysis undertaken. For the 2009/10 winter the number of emergency hospital admissions for falls on snow and ice were plotted by age and gender. The inpatient costs of admissions in the 2009/10 winter for falls on snow and ice were calculated using Healthcare Resource Group costs and Admitted Patient Care 2009/10 National Tariff Information.

**Results:**

The number of emergency hospital admissions due to falls on snow and ice varied considerably across years; the number was 18 times greater in 2009/10 (N = 16,064) than in 2007/08 (N = 890). There is an exponential increase [Ln(rate of admissions) = 0.456 - 0.463*(mean weekly temperature)] in the rate of emergency hospital admissions for falls on snow and ice as temperature falls. The rate of admissions in 2009/10 was highest among the elderly and particularly men aged 80 and over. The total inpatient cost of falls on snow and ice in the 2009/10 winter was 42 million GBP.

**Conclusions:**

Emergency hospital admissions for falls on snow and ice vary greatly across winters, and according to temperature, age and gender. The cost of these admissions in England in 2009/10 was considerable. With responsibility for health improvement moving to local councils, they will have to balance the cost of public health measures like gritting with the healthcare costs associated with falls. The economic burden of falls on snow and ice is substantial; keeping surfaces clear of snow and ice is a public health priority.

## Background

Prolonged snow and icy conditions resulting in slippery roads and pavements are a problem for everyone because few people are able to avoid venturing outside for any sustained length of time. In the United Kingdom (UK), snow is not an unusual occurrence, but neither is it commonly a major concern. The winter of 2009/10, however, was different; according to the Meteorological Office, the mean temperature for the UK was 1.6 degrees centigrade (°C), making it the coldest winter for 30 years, with significant snow falls occurring widely across the country from mid-December to the end of February [1].

The duration and severity of the cold temperatures during the winter of 2009/10 resulted in increased demand for salt to grit the roads and pavements. According to the Local Highways Authority, the cost of treating the roads during 2009/10 was 49% higher than it cost in the winter of 07/08, but despite the additional gritting activity, stockpiles became depleted in many areas of the UK and many surfaces were left untreated [2]. While some people found the prolonged icy conditions a welcome opportunity to stay away from school and work, for others they represented a serious health hazard. In particular, icy conditions are known to increase the likelihood of experiencing a fall [3], with accidents resulting in various injuries, most commonly fractures and back injuries [4-6]. Consequently, one hospital in the South West of England, for example, reported that, in the first two weeks of January 2010, the number of admissions to the emergency ward because of falls due to slipping on snow and ice, and the number of trauma operations performed, had doubled, costing an additional 227,500 GBP [7].

There has been little research conducted in the UK into the relationship between winter weather conditions and falls, and more specifically, the impact of prolonged cold temperatures. The first aim of this study was to describe the relationship between winter temperature and the number and rate of emergency admissions to hospital due to falls on snow and ice in England. Secondly, for the 2009/10 winter, we aimed to identify the age and gender of those most at risk of being admitted to hospital for a fall due to snow and ice. The final aim was to calculate the costs associated with emergency hospital admissions due to falls on snow and ice in England during the 2009/10 winter. Winter is defined as 1^st ^December to 28^th^/29^th ^February [1].

## Methods

Data were extracted from the Hospital Episode Statistics (HES) dataset which records inpatient care from National Health Service (NHS) hospitals across England. Within this dataset, a unit of care (a finished consultant episode, FCE) equates to the period a patient spends under the care of a single hospital consultant and several FCEs may make up a continuous period of inpatient care, or spell. The number of emergency hospital admissions for falls due to snow and ice were derived from HES for winters 2005/06 to 2009/10. More specifically, completed FCE records which were the first episode in a spell of care, and had an admission date between 1^st ^December and 28^th^/29^th ^February, were extracted where any of the 20 diagnosis fields contained the International Classification of Disease (ICD-10) code 'W00' which has the description 'external cause of morbidity and mortality: fall on same level involving ice and snow' [8]. Using the extracted data, the following were calculated separately for winters 2005/06 to 2009/10: 1) the total number of emergency admissions from falls due to ice and snow in England, and 2) the number of emergency admissions for falls due to snow and ice per week, per Government Office Region (GOR, using the patient's postcode of residence to assign each admission to a region).

Regional temperature data are available from the UK's Meteorological Office. Temperature data are not recorded at the GOR level but are collected by television regions which cover approximately the same areas as the nine GORs (the West Country and West television regions were amalgamated and the mean temperature was derived in order that temperature data corresponded with the South West GOR, while the Central television region covered both East Midlands and West Midlands GORs). The UK's Meteorological Office provided: 1) mean winter temperature data for England for winters 2005/06 to 2009/10, and 2) mean weekly temperature data for England television regions for winters 2005/06 to 2009/10.

Mean England temperatures for winters 2005/06 to 2009/10 were plotted against the total number of emergency hospital admissions in England from falls due to snow and ice for the corresponding winter. Secondly, for each Government Office Region separately (in order to control for regional temperature variations), mean weekly temperature (winters 2005/06 to 2009/10 separately) were plotted against the corresponding rate of weekly emergency hospital admissions for falls on snow and ice and showed an exponential relationship. Taking the natural logs of the rates of weekly emergency hospital admissions for falls on snow and ice produced a strong linear relationship with mean weekly temperature, evidenced by R = 0.708, therefore a linear regression analysis was implemented using logs of the rates of weekly emergency admissions for falls on snow and ice as the dependent variable and mean temperature as the independent component. The underlying assumptions of normality and equal variance of the errors for a linear regression model were checked and validated with no evidence to the contrary. Thirdly, for the 2009/10 winter, the rate of emergency hospital admissions for falls due to snow and ice in England was plotted by age and gender.

The emergency hospital admission costs of falls due to snow and ice were calculated for the 2009/10 winter using Healthcare Resource Group (HRG) codes, which are used to calculate the cost of care by applying the Department of Health's Payment by Results (PbR) system. PbR was first introduced in the NHS in 2003/04 to improve the fairness and transparency of hospital payments and to stimulate provider activity and efficiency. Rather than relying on locally negotiated contracts based on local prices providers are paid for the number and type of patients treated in accordance with national rules and a national tariff. The development of HRGs support PbR by providing a classification framework that represents current clinical practice. In their most basic form, HRGs are groups of ICD-10 diagnoses and operational procedures that have similar resource implications, and which offer a means of determining fair and equitable reimbursement for care services delivered by providers [9].

The NHS Information Centre's current classification HRG4 [10], and a corresponding software application, the 2009/10 Local Payment Grouper [11] were used to calculate costs of falls. All (first and subsequent) completed emergency FCEs due to falls on snow and ice with an admission date between 1^st ^December 2009 and 28^th ^February 2010 were extracted and entered into this software. Of the 16,458 FCEs input, 734 (4.5%) were identified as invalid due to coding errors in the original HES data and these were removed from the analysis; the valid 15,724 FCEs were grouped by the software into 15,331 spells.

The mandatory Admitted Patient Care 2009/10 National Tariff Information [12] was then applied to the dominant HRG and a corresponding cost generated for each spell. National tariff prices are the national average NHS costs of procedures, adjusted for inflation. They are derived from the reference costs that all NHS providers calculate and submit to the Department of Health as part of their annual accounting process. Reflecting the actual cost of procedures - or as close to the actual cost as systems allow - rather than a budgeted cost, these actual costs include a share of all the support functions and overheads of the trust providing the treatment.

Finally, the cost of the spells were adjusted in accordance with the typical number of bed days for the treatment taking place; outlier cases, which fall beyond the upper or lower 'trim points' set within the national tariff were reduced or increased based on long or short stays respectively. More specifically, where the spell length exceeded the trim point specific to their HRG, the cost of additional bed days were added to the original cost of the spell. Conversely, the short stay tariff was used to adjust the costs for stays which were less than two days where the average HRG length of stay is longer. This short stay tariff is revised annually and reflects the high amount of resources used in the first day of care.

## Results

**Figure 1:**
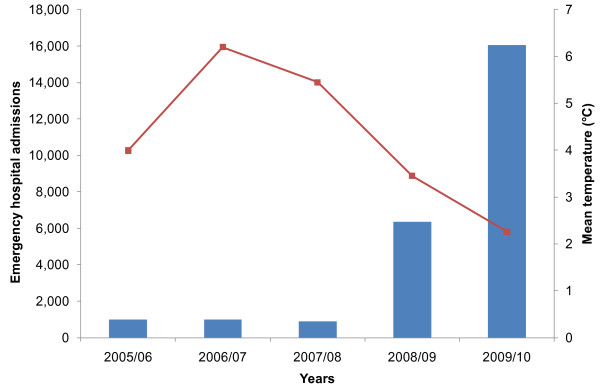
**Number of emergency hospital admissions for falls on snow and ice and mean temperature, England winters**. Winter is defined as 1^st ^December to 28^th^/29^th ^February.

The number of emergency admissions to hospital in England due to falls on snow and ice for years 2005/06 to 2009/10 are shown in Figure 1, along with the corresponding mean winter temperature. The lowest number of emergency admissions, 890, occurred in 2007/08, while the number of admissions in 2009/10 was 16,064; the number of emergency hospital admissions for falls on snow and ice was therefore 18 times greater in 2009/10 than in 2007/08.

**Figure 2:**
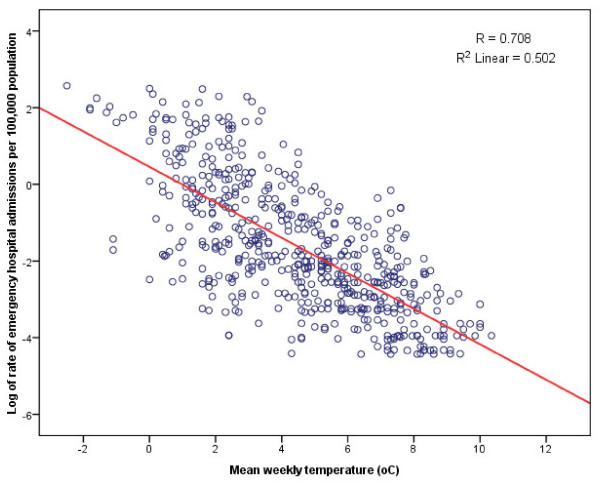
**Logs of the weekly rates of emergency hospital admissions for falls on snow and ice against temperature, England winters**. Winter is defined as 1^st ^December to 28^th^/29^th ^February, however here dates are extended just outside this date range in order to include full weeks of data. Rates are calculated as the number of admissions per 100,000 of population. Data include the following winters: 2005/06 to 2009/10.

The results show that about half the variance in the logs of the rates of emergency hospital admissions for falls on snow and ice is explained by the model, evidenced by R squared = 0.502. Inspecting the coefficient for mean temperature, B = -0.463 (t = -23.635, P < 0.001) shows there is a significant negative correlation with log of emergency hospital admissions for falls on snow and ice as expected, which means as the temperature drops, there is a corresponding exponential increase in the weekly rate of admissions [Ln(rate of admissions) = 0.456 - 0.463*(mean weekly temperature), Figure 2]. When transforming the prediction to an estimate of the weekly rate of emergency hospital admissions for falls on snow and ice, for temperatures below 1°C, the prediction is generally underestimating the true rate suggesting that the exponential increase may be stronger at lower temperatures.

**Figure 3:**
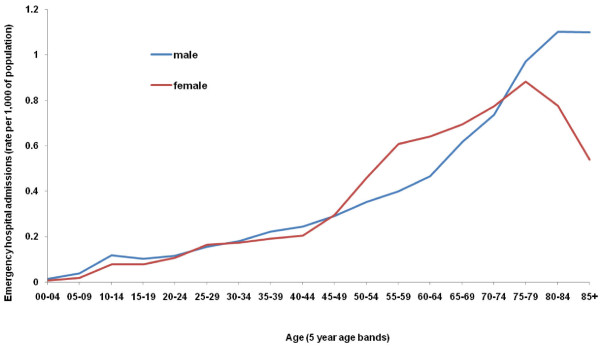
**Rate of emergency hospital admissions for falls on snow and ice by age and gender, England winter 2009/10**. Winter is defined as 1^st ^December to 29^th ^February. Rates are calculated as the number of admissions per 1,000 of population.

The rate of emergency hospital admissions due to falls on snow and ice during the winter of 2009/10 varied by age and gender (Figure 3). The rate was higher among females than males between the age groups 45 to 49 and 70 to 74; the rate among women aged 55 to 59 was 0.61 per 1,000 of population, while the corresponding rate among men was 0.40 per 1,000 of population. The rate among women declines after the age group 75 to 79, while the rate among men rises and plateaus; consequently at the age of 85 and over, the rate of emergency hospital admissions among women is 0.54 per 1,000 of population, while for men the rate is 1.10 per 1,000 of population.

The total cost for England of emergency hospital admissions due to falls on snow and ice was 42,318,960 GBP; 23,137,182 GBP, or 54.7% of the total, was for the cost of emergency hospital admissions for women (Table 1).

**Table 1:** Cost of emergency hospital admissions for falls on snow and ice by age and gender, England winter 2009/10

Age group (years)	Cost (GBP)		
	
	Males	Females	Total
20 or below	797,129	543,961	1,341,090

21 to 35	1,800,935	1,828,986	3,629,921

36 to 50	3,519,232	3,414,626	6,933,858

51 to 65	4,878,114	7,594,261	12,472,375

Over 65	8,186,368	9,755,348	17,941,716

**Total**	**19,181,778**	**23,137,182**	**42,318,960**

Winter is defined as 1^st ^December to 29^th ^February. Costs are calculated using Healthcare Resource Group costs and Admitted Patient Care 2009/2010 National Tariff Information.

## Discussion

Here we show that the number of emergency hospital admissions in England due to falls on snow and ice varies considerably by year; in 2007/08 there were 890 hospital admissions in England, while there were 16,064 in 2009/10 (Figure 1). There is an inverse relationship between the rate of weekly emergency hospital admission due to falls on snow and ice and weekly temperature and this relationship is exponential (Figure 2). For temperatures below 1°C the prediction underestimates the true rate suggesting that the exponential increase in the rate of emergency admissions for falls on snow and ice may be stronger for lower temperatures. The association between the rate of emergency hospital admissions due to falls on snow and ice and temperature would likely be even stronger if daily temperature had been used and the effects of short fluctuations in temperature that can occur during a week had been captured. However, the focus of this paper is the impact of the prolonged cold temperatures lasting more than a couple of days; circumstances which are relatively unusual in England and which we hypothesised would result in increased admissions for falls on snow and ice because the infrastructure is less able to cope than during the more common occurrence of a day or so of cold temperatures.

The winter of 2009/10 was particularly cold [1] and in many areas of the UK, pavements and roads were left untreated allowing snow and ice to accumulate [2]. During this period, the highest rate of emergency hospital admissions due to falls on snow and ice were among the elderly, and particularly among men aged over 80 (Figure 3). Interestingly, the relationship between gender and age and the rate of emergency hospital admissions for falls presented here was similar to previous work conducted in Sweden, which also showed a higher rate of admissions among women than men aged 50 and 79, followed by a higher rate among men aged over 80 [4]. Implementing measures such as organising snow clearing and assistance with shopping for elderly people would decrease their exposure to falls, while the use of anti-slipping devices for shoes and personal protection devices like hip padding could also play an important preventative role [4]. Research conducted in the USA on all falls among people aged 65 and over, demonstrated that fall related medical expenditure is two to three times higher for a woman than a man, with authors hypothesising that this discrepancy was due to higher costs of osteoporotic fractures, which disproportionately affect women [13].

Here we show that the costs for hospital trusts in England of treating emergency admissions due to falls on snow and ice during this three month period was 42 million GBP. The total cost of these accidents to the health services is likely to be much higher. Firstly, the costs presented here do not include the cost of treating people who attended Accident and Emergency departments but who were not admitted to hospital, nor the costs of people who contacted primary care services like their GP, pharmacist or 'walk in' centre for treatment. Secondly, the costs detailed here do not include longer term health and social care costs. Among older people, for example, falls are often associated with hip fractures which frequently lead to loss of independence and disability requiring extensive rehabilitation (in NHS outpatient departments) and nursing home admission [13,14]. Indeed a study in the UK estimated that the healthcare costs associated with a typical hip fracture in 2000 were approximately 25,000 GBP with long stay residential care accounting for 80% of this cost [14]. Finally, the costs do not include falls on snow and ice that occurred outside of December to February, nor do they include wider societal costs as a result of sickness absence from work.

Worldwide, falls are the second leading cause of deaths from accidental or unintentional injury [15], but snow and ice injuries are different from other major accidents in that they are predictable and preventable. A key aspect of fall prevention strategies is creating safe environments [15] and the most important aspect for preventing falls involving snow and ice is clearing pavements [5]. Following the 2009/10 winter, much discussion ensued about the wisdom of stockpiling large amounts of salt to clear roads from snow and ice and the cost of this if it was not used. Discussions also focused on the costs to the economy of lost revenue from business which had to close because employees could either not make it to work or because of childcare issues due to school closures. The financial cost of falling on snow and ice received little attention.

Within the context of Government proposals for massive changes to the way in which the health service is delivered in England, responsibility for health improvement will be transferred from primary care trusts to local authorities [16], with local authority based health and wellbeing boards taking on the responsibility of joining up health and social care commissioning and partnership working [17]. Consequently, in the future, local authorities will need to balance the costs of public health prevention measures, such as gritting the roads and pavements, and indeed the costs of stockpiling salt in preparation of this, with the economic and healthcare costs associated with falls due to snow and ice. Such decisions will be made all the more difficult in the coming winters because Council Tax is frozen for at least a year and possibly even longer [18].

A significant focus of the new UK coalition Government is to encourage social responsibility and volunteering and people coming together to help one another and improve their communities [18]. The Local Government Association has reiterated these sentiments, believing that a joined up approach to snow clearing that encourages the public to take a more active role would help to ensure that more footpaths and cycle paths remain clear [2]. Indeed a newspaper report suggests that Knowsley council in Merseyside, in the North West of England, has already suggested that they will advocate that the public and businesses keep pavements clear of ice [19]. In other European countries, like Germany, Austria and Switzerland, regulation is already in place that requires citizens to keep their pavements clear of snow. In the US state of Minneapolis, snow must be removed within 24 hours of the snow fall ending, while in Boston, fines can reach up to $250 for every day that the snow remains [20]. While such initiatives seem sensible, a significant obstacle to this approach in the UK is public concern about litigation should an injury occur on an area that they have cleared [2]. In response to these concerns, and wider unease about the growth of a compensation culture in the UK, a recent Government review of the operation of health and safety laws identified the publication of snow clearing guidelines as a key milestone for immediate consideration [21]. However, clarifying the legal position may not be enough to encourage the public to act altruistically towards other members of their community; of the 14,800 excess deaths in Paris during the heat wave of 2003, 19% occurred at home [22], suggesting a lack of family or community support for those in high risk populations.

The UK population is ageing and the cost of falls to the NHS is expected to rise [14], while the cost of hip fractures is predicted to double between 2007 and 2050 [23]. The economic burden of falls underscores the need for effective prevention interventions, including those that focus upon preventing falls due to snow and ice. The media (for example meteorological forecasts) can be effective in communicating health protection messages to the public [22] but the information must be meaningful and presented in a manner which supports those people most at risk to make sensible decisions.

## Conclusions

In England, the number of emergency hospital admissions due to falls on snow and ice varies considerably across winters. We show that the weekly rate of emergency hospital admissions due to falls on snow and ice is inversely related to the mean weekly temperature, with an exponential increase in admissions occurring as the temperature falls. The winter of 2009/10 was characterised by low temperatures for a prolonged period of time. Many areas of the country were unprepared for these conditions, meaning that grit supplies became depleted and many surfaces were left untreated. The rate of emergency hospital admissions for falls on snow and ice during the winter of 2009/10 was particularly high for the elderly. Here we estimate that in England, the cost of emergency admissions to hospital for falls on snow and ice between 1^st ^December 2009 and 28^th ^February 2010 was 42 million GBP. However, the true healthcare costs will be considerably higher. With responsibility for health improvement being transferred from primary care trusts to local authorities, in the future local councils will have to balance the costs of public health measures such as gritting the roads and pavements, with the economic and healthcare costs associated with falls due to snow and ice. The economic burden of falls due to snow and ice necessitates effective prevention interventions and, with December 2010 to January 2011 also being hit by unusually prolonged cold temperatures and associated snow fall [24], the necessity of keeping surfaces clear of snow and ice should be considered a public health priority.

## List of abbreviations

FCE: finished consultant episode; GP: general practitioner; HES: hospital episode statistics; HRG: Healthcare Resource Group; ICD: International Classification of Disease; NHS: National Health Service; PbR: Payment by Results.

## Competing interests

The authors declare that they have no competing interests.

## Authors' contributions

CB conceived of the study and its design, and drafted the manuscript. SW carried out data extraction, calculated the costs and participated in drafting the manuscript. MR conducted data analysis and participated in drafting the manuscript. JM participated in data analysis and in drafting the manuscript. KM participated in data analysis and in drafting the manuscript. IJ conducted the statistical analysis and participated in drafting the manuscript. MB participated in the design of the study and drafting the manuscript. CP conceived of the study, and participated in drafting the manuscript. All authors read and approved the final version.
